# Integrating physiological data with the conservation and management of fishes: a meta-analytical review using the threatened green sturgeon (*Acipenser medirostris*)

**DOI:** 10.1093/conphys/coz035

**Published:** 2019-06-28

**Authors:** Essie M Rodgers, Jamilynn B Poletto, Daniel F Gomez Isaza, Joel P Van Eenennaam, Richard E Connon, Anne E Todgham, Alicia Seesholtz, Joe C Heublein, Joseph J Cech, John T Kelly, Nann A Fangue

**Affiliations:** 1 Wildlife, Fish and Conservation Biology, University of California Davis, One Shields Ave., Davis, CA, USA; 2 School of Natural Resources, University of Nebraska-Lincoln, 3310 Holdrege St., Lincoln, NE, USA; 3 School of Biological Sciences, The University Queensland, Brisbane, QLD, Australia; 4 Department of Animal Science, University of California Davis, One Shields Ave., Davis, CA, USA; 5 Department of Anatomy, Physiology and Cell Biology, University of California Davis, One Shields Ave., Davis, CA, USA; 6 California Department of Water Resources, Industrial Blvd., West Sacramento, CA, USA; 7 NOAA National Marine Fisheries Program, West Coast Region, Capital Mall, Sacramento, CA, USA; 8 Fisheries Branch, California Department of Fish and Wildlife, Sacramento, CA, USA

**Keywords:** Conservation physiology, data synthesis, ecophysiology, fish conservation, fish management, stressors

## Abstract

Reversing global declines in the abundance and diversity of fishes is dependent on science-based conservation solutions. A wealth of data exist on the ecophysiological constraints of many fishes, but much of this information is underutilized in recovery plans due to a lack of synthesis. Here, we used the imperiled green sturgeon (*Acipenser medirostris*) as an example of how a quantitative synthesis of physiological data can inform conservation plans, identify knowledge gaps and direct future research actions. We reviewed and extracted metadata from peer-reviewed papers on green sturgeon. A total of 105 publications were identified, spanning multiple disciplines, with the primary focus being conservation physiology (23.8%). A meta-analytical approach was chosen to summarize the mean effects of prominent stressors (elevated temperatures, salinity, low food availability and contaminants) on several physiological traits (growth, thermal tolerance, swimming performance and heat shock protein expression). All examined stressors significantly impaired green sturgeon growth, and additional stressor-specific costs were documented. These findings were then used to suggest several management actions, such as mitigating salt intrusion in nursery habitats and maintaining water temperatures within optimal ranges during peak spawning periods. Key data gaps were also identified; research efforts have been biased towards juvenile (38.1%) and adult (35.2%) life-history stages, and less data are available for early life-history stages (embryonic, 11.4%; yolk-sac larvae, 12.4%; and post yolk-sac larvae, 16.2%). Similarly, most data were collected from single-stressor studies (91.4%) and there is an urgent need to understand interactions among stressors as anthropogenic change is multi-variate and dynamic. Collectively, these findings provide an example of how meta-analytic reviews are a powerful tool to inform management actions, with the end goal of maximizing conservation gains from research efforts.

## Teaser

A wealth of data exists on the ecophysiological constraints of many fishes, but much of this information is underutilized in recovery plans due to a lack of synthesis. We used the imperiled green sturgeon as an example of how a quantitative synthesis of physiological data can inform conservation plans.

## Introduction

The loss of biodiversity of fishes is occurring worldwide, with many fish populations in serious decline (Moyle and Leidy, 1992; Dudgeon *et al.*, 2006). These losses are particularly acute in areas where anthropogenic stressors have led to habitat loss and degradation or changes in environmental variables (Marchetti and Moyle, 2001; Wenger *et al.*, 2011; Hooper *et al.*, 2012). As the need to effectively manage and conserve fishes has become more urgent, there has been a corresponding call for a greater understanding of the physiological ecology and behaviour of species in question. The profound lack of available detailed physiological and behavioural data has been underscored by fish population declines, and the data gaps often present a barrier to moving forward with new management and conservation actions. Similarly, managers are often unaware of what data currently exist and how this information could potentially be useful in mitigating or preventing further population declines. In the fields of fisheries science as well as experimental fish physiology and conservation biology, a call for greater integration of physiological information with fisheries management has been made in recent years (Wikelski and Cooke, 2006; Horodysky *et al.*, 2015).

Physiology is extremely well suited to inform conservation efforts, including detailed information about environmental tolerances and optima (e.g. temperature, salinity, water velocity, turbidity, etc.), growth rates under different conditions, sensitivity to environmental contaminants and susceptibility to predation by invasive species (Cooke *et al.*, 2013). This information is critical for species persistence and an awareness of the need for it has been reflected in recent status reports and assessments issued by conservation agencies such as the National Oceanographic Atmospheric Administration’s National Marine Fisheries Service (NMFS). Collaborations between physiologists and fisheries managers have proven successful at more effectively managing fish populations for decades, though the emergence of a discipline to support this integration is a more recent development (Cooke *et al.*, 2013). For example, the inclusion of water temperature as a critical abiotic factor affecting the growth, survival and distribution of fishes in management plans is long-standing (U.S. Fish and Wildlife Service, 1995). Much of our understanding of how fishes respond to both acute and gradual changes in temperature is derived from physiological and behavioural studies conducted in the laboratory. A recent recovery plan released by NMFS included elevated temperatures as a key factor affecting populations of threatened and endangered Chinook salmon (*Oncorhynchus tshawytscha*) in California, citing physiological stress, increased energetic costs, reduced growth rates and higher incidence of disease as physiological mechanisms contributing to population declines (NMFS, 2014). Indeed, numerous water temperature control projects, such as regulated flow regimes or structured water releases from reservoirs, are currently in effect around the world to help create thermal habitat that will promote the survival and persistence of species of management concern. Similarly, physiology has helped mitigate direct human-induced mortality on fishes, including the regulation of fishing pressure based on predictive estimates made by integrating physiological and environmental data. Cooke *et al.* (2012) reviewed the numerous physiological studies that contributed to the regulation and management of Pacific salmon (*Oncorhynchus*) fisheries, including those that affected management of some imperiled populations of Fraser River sockeye salmon (*O. nerka*). As fish populations continue to decline and conservation and management plans become more detailed, the need to provide quantitative syntheses of existing data is crucial.

Although there are examples of physiological data being incorporated into biological opinions, recovery plans and assessments of conservation actions, this integration is often limited in scope or depth. As such, the need for a more systematic and quantitative approach to integrate physiological data into conservation plans is required. This includes not only a way to utilize the already existing data but also a process to identify knowledge gaps, with the overall goal of increasing the efficacy of future management and research efforts. Here, we used the imperiled green sturgeon (*Acipenser medirostris*, Ayres, 1854) as an example of how a quantitative synthesis of physiological data can inform conservation plans and future research actions. Green sturgeon are long-lived, anadromous fish inhabiting waters of the Pacific Northwest region of North America. Sturgeons are members of the subclass *Chondrostei* and family *Acipenseridae*, a group of evolutionarily ancient fish that diverged from the more-derived teleosts over 200 million years ago (Moyle, 2002). Green sturgeon are slow to reach sexual maturity compared to most fishes (typically 15–17 years old and 1.5–1.6 m total length, TL; Erickson and Webb, 2007) and have a spawning periodicity of 3–5 years (Moyle, 2002)—characteristics that make them particularly vulnerable to population declines. The green sturgeon population is comprised of two genetically distinct population segments (DPSs; Israel *et al.*, 2004) that differ in spawning ground locations. The northern DPS spawns principally in the Rogue River in Oregon and the Klamath River in California, though both the Umpqua and Eel Rivers may contribute to the spawning population (Erickson *et al.*, 2002). The southern DPS spawns primarily in mainstem Sacramento River in California (Israel *et al.*, 2004; Israel and Kimley, 2008), though recent evidence documented spawning in the Feather River and the Yuba River as well (Seesholtz *et al.*, 2015). Due to the restricted spawning grounds of the southern DPS, as well as habitat degradation and evidence of declining population numbers, the southern DPS was listed by the National Marine Fisheries Division of the National Oceanic and Atmospheric Administration in 2006 as ‘threatened’ under the federal Endangered Species Act of 1973. The northern DPS remains unlisted, although it is considered a ‘species of concern’ under federal designation.

The drivers of green sturgeon population declines are unclear but key threatening processes are thought to be at play. Early life stages (embryos, larvae and young juveniles) are restricted to heavily altered riverine habitats and face multiple anthropogenic threats, such as the intrusions of contaminants and salt, the introduction of exotic species and the installation of dams and water diversion pipes (NMFS, 2010, 2018). Conversely, there are few known threats (besides fisheries capture and ocean acidification) to green sturgeon during their marine life history phase, and life-stage-specific management guidelines are required for this species. A wealth of data exists on the ecophysiological constraints of green sturgeon, but much of this information is underutilized in recovery plans due to a lack of synthesis. Therefore, this review and meta-analysis synthesized data on green sturgeon ecophysiology published in peer-reviewed journals. A meta-analytical approach was opted to summarize the effects of prominent stressors [i.e. elevated temperatures, contaminants, salinity and low food availability (food restriction)] on several physiological traits [e.g. growth, thermal tolerance, swimming performance and heat shock protein (Hsp) expression] using quantitative methods. The aim of this study was threefold: (i) to characterize the variety of research output on green sturgeon, in terms of the field of study (e.g. conservation physiology, movement ecology, etc.), type of study (experimental, field or theoretical), life-history stage examined and number of concurrent stressors examined; (ii) to quantify the overall effect of prominent stressors on key physiological traits; and (iii) to highlight areas where sufficient data exist to inform recovery plans and identify knowledge gaps to direct future research efforts. Collectively, the information derived here will provide an example of how a quantitative synthesis of physiological data can inform conservation plans and future research actions.

## Materials and Methods

### Review protocol

A Web of Science (Thompson Reuters) search was performed to find publications containing green sturgeon or *Acipenser medirostris* as key words. These publications were refined to only include peer-reviewed research papers. The following data were extracted from all publications: scientific journal, publication year, geographic location, type of study (experimental, field or theoretical), stressor of interest, population (southern or northern DPS), life-history stage examined (embryos, yolk-sac larvae, larvae, juveniles, sub-adults or adults) and age (days post-hatch). For studies examining the effects of stressors on physiological responses the duration of stressor exposure (acute ≤ 2 weeks < chronic) was noted, along with the number of concurrent stressors examined (i.e. single- or multi-stressor study).

### Meta-analysis

Additional metadata were extracted from studies examining the effects of elevated temperatures, salinity, food restriction and contaminants on green sturgeon physiology. Only manipulative experiments with controls were included. Studies on the impact of movement barriers (e.g. dams and water diversion pipes) on green sturgeon were generally not manipulative and measured incomparable response variables (e.g. movements of free-ranging individuals versus laboratory entrainment rates in diversion pipes), and data were insufficient for quantitative analysis. However, movement barriers present a major threat to green sturgeon (NMFS, 2010, 2018), so a qualitative assessment of movement barriers was included in this review. To enable effect size calculations, studies were only included if means, sample sizes and variance (standard error, standard deviation or confidence interval) were reported. In instances where means and variance were not reported in text, data were extracted from figures using ImageJ (v. 1.51 k, National Institute of Health, USA, http://imagej.nih.gov.i)—a validated and commonly used method (Schneider *et al.*, 2012; Schindelin *et al.*, 2015; Foley *et al.*, 2018). For each stressor the following definitions for control groups were used: elevated temperatures (control = 16–19°C; treatment > 19°C); salinity (control < 3 ppt; treatment > 3 ppt), food restriction (control = unrestricted, *ad libitum*; treatment = restricted) and contaminants (control = no contaminant; treatment = contaminant). The effects of the stressors on the following response variables were examined: growth, Hsp expression, deformity rates, hatching success, swimming performance (*U*_crit_), critical thermal maxima (CTMax), mortality rates, whole-body contaminant burden, plasma osmolality and muscle water content. A minimum of four data points for each trait were required to perform analyses, and response variables that did not meet this criterion were considered data deficient (D.D.). Effect sizes were calculated as the log-response ratio (*lnRR*),

\begin{equation*} LnRR = ln \left(\frac{\overline{X}_T}{\overline{X}_C}\right) = ln \big(\overline{X}_{T} \big) - ln \big(\overline{X}_{C} \big), \end{equation*}

where $\overline{X}_{T}$ and $\overline{X}_{C}$ are the mean responses from the treatment and the control groups, respectively. The variance (*v*) of each *ln-*transformed response ratio was also calculated,

\begin{equation*} v = \frac{\big({SD}_T\big)^2}{N_{T\ {\overline{X}}_T^2}}+\frac{{\big({SD}_C\big)}^2}{N_{C\ {\overline{X}}_C^2}},  \end{equation*}

where *SD* and *N* are the standard deviation and sample size of the treatment and the control groups. Log response ratios were chosen over other methods because response ratios calculate the proportional change between the control and the treatment groups. Log response ratios are also robust to small sample sizes, less sensitive to data of different units and normalize data that are not normally distributed (Lajeunesse, 2011; Viechtbauer, 2010). Separate random effects models were used to calculate the overall effect size for each stressor (elevated temperature, salinity, food restriction and contaminants). Random effects models account for within- and between-study variation, and effect sizes were considered significant if 95% confidence intervals did not overlap with zero. Log response ratios and variances were calculated using the *escalc* function of the *metafor* package (Viechtbauer, 2010) in R Studio (version 1.0.136).

### Publication bias

Contour-enhanced funnel plots of observed effect sizes were used to test for publication bias using the *funnel* function in *metaphor* in R Studio (version 1.0.136). Regression tests were used to test for asymmetry in the funnel plot using the *regtest* function (Egger *et al.*, 1997), and publication bias was inferred if the regression slope was significantly different from zero. If asymmetry was detected, the *trimfill* function was used to determine the number of studies needed to obtain symmetry. The *trimfill* function assesses the sensitivity of the results to publication bias by ‘filling in’ the number of missing studies to obtain symmetry (Møller and Jennions, 2001). Lastly, Rosenberg’s fail-safe numbers were calculated and represent the number of papers that would need to be published with non-significant results to change the mean effect size to a non-significant result (Rosenberg, 2005). Fail-safe numbers are considered robust to publication bias when larger than 5*n* + 10 (where *n* is the number of studies already included in the meta-analysis; Rosenberg, 2005).

## Results

**Figure 1:**
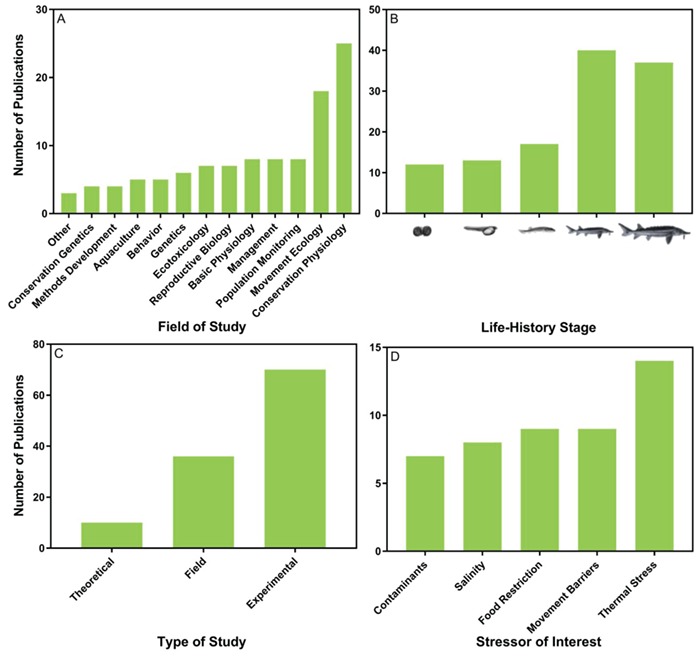
Summary of the research output on green sturgeon, (*Acipenser medirostris*) in terms of (A) field of study, (B) life-history stage examined, (C) type of study and (D) type of stressor examined

A total of 105 publications were found containing green sturgeon or *Acipenser medirostris* as key words (Supplementary material). Research on green sturgeon spans multiple disciplines (Fig. 1A), with a primary focus on conservation physiology (23.8%), movement ecology (17.1%), and basic physiology (7.6%). Mixtures of experimental, field and theoretical research approaches have been employed (Fig. 1C). Data on juvenile (38.1%) and adult (35.2%) life-history stages of green sturgeon are predominant, and less data are available for the early life-history stages (embryonic, 11.4%; yolk-sac larvae, 12.4%; and post yolk-sac larvae, 16.2%; Fig. 1B). The effects of stressors on physiological responses have included both acute (38.8%) and chronic (61.2%) responses; however, most data have been collected from single stressor studies and only 6 out of 70 experimental studies (~8.6%) examined the combined effects of multiple stressors. Thermal stress has received the majority of investigation, although research efforts (# publications) have been distributed evenly across stressors of interest (i.e. contaminants, salinity, food restriction and movement barriers) (Fig. 1D). All experimental studies were conducted on the northern DPS.

### Meta-analysis

#### Elevated temperatures

**Figure 2:**
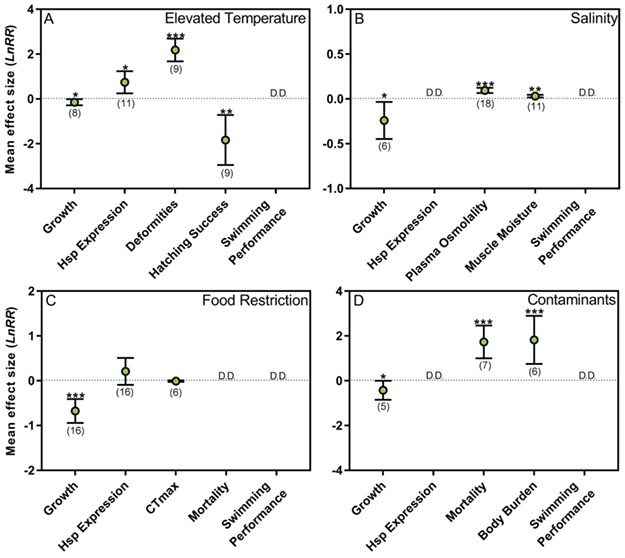
The impact of (A) elevated temperatures, (B) salinity, (C) food restriction, and (D) contaminants on several physiological traits in green sturgeon (*Acipenser medirostris*) from random effects meta-analyses. Data are presented as mean effect size (log ratio response, *LnRR*) and 95% confidence intervals. Sample sizes in each analysis are included in parentheses. The horizontal zero (dotted) line indicates no effect, and mean effect sizes are considered significant when 95% confidence intervals do not overlap with the zero line as denoted by an asterisks (^*^P < 0.05, ^**^P < 0.01, ^***^P < 0.001). D.D. represents response variables with less than four data points for comparison. See main text for abbreviations.

The results of the random effects model show that elevated temperatures significantly reduce growth and hatching success and increase the incidence of larval deformities (Fig. 2A). Exposure to elevated temperatures also increases the expression of Hsp. There were insufficient data to assess the impact of elevated temperatures on swimming performance. Regression tests indicated that there was no significant asymmetry in the random effects models for growth, Hsp expression and deformity rate. However, significant asymmetry was detected in response to hatching success (Supplementary Table I). The *trimfill* method revealed that two data points were augmented in order to obtain symmetry. Rosenberg’s fail-safe numbers were robust (ranging from 489 to 4439) for all response variables, indicating that a large number of non-significant results are required to alter the conclusions (Supplementary Table I).

#### Salinity

On average, exposure to elevated salinity levels negatively affects growth (Fig. 2B). Further, plasma osmolality and muscle moisture are significantly increased in response to salinity exposure. There were insufficient data to assess the impacts of salinity on expression of Hsp and swimming performance. Asymmetry in the regression tests for growth, plasma osmolality and muscle moisture were not detected, indicating no publication bias (Supplementary table I). Rosenberg’s fail-safe numbers are robust for the plasma osmolality (1335) and muscle moisture (377) datasets, but not for growth (12).

#### Food restriction

Growth was significantly reduced by food restriction (Fig. 2C), but food restriction does not affect the upper thermal tolerance (CTMax) or expression of Hsp. Although mortality rates tend to increase and swimming performance decrease with food restriction, there were insufficient data to assess its impact. Publication bias was not detected for the growth data set, as indicated by the regression test and Rosenberg’s fail-safe number (498; Supplementary table I).

#### Contaminants

Exposure to environmental contaminants (carbaryl, methylmercury and selenium) significantly reduced growth and increased the body burden and mortality rates of green sturgeon (Fig. 2D). There were insufficient data to assess the impact of contaminants on the swimming performance and expression of Hsp. Regression tests indicated that there was no significant asymmetry in the random effects models for growth, mortality and body burden (Supplementary table I). Rosenberg’s fail-safe numbers were robust to publication bias for the mortality (167) and body burden (244) datasets, but not for growth (5).

## Discussion

Green sturgeon research has been multi-disciplinary, with a primary focus on conservation physiology. The meta-analysis revealed that all examined stressors significantly impaired green sturgeon growth, and additional stressor-specific costs were identified. These findings can be used to tailor management actions to meet the ecophysiological requirements of green sturgeon and plan future research efforts. Here we review and discuss the impact of prominent stressors (i.e. thermal stress, food restriction, elevated salinity, contaminants and movement barriers) on each green sturgeon life-history stage, identify knowledge gaps and suggest science-based management actions.

### Thermal stress

As ectotherms, green sturgeon body temperature is closely tied to the external environment and thermal perturbations are particularly salient due to the thermal sensitivity of molecular, cellular and metabolic processes (Hochachka, 1967; Hochachka and Somero, 1968). The thermal sensitivity of key traits, such as swimming performance, can be described using thermal performance curves (TPCs), where an ectotherms’ performance is modelled as a function of environmental/body temperature (Huey and Stevenson, 1979; Huey *et al.*, 2012). Ectotherm TPCs are generally bell shaped; performance is optimized within a narrow thermal window (Topt) and performance decrements are observed as temperatures deviate towards low and high ends. Performance is entirely reduced once temperatures reach low and high critical limits—critical thermal minima (CTMin) and CTMax, respectively. However, the biological relevance of CTMin can be limited in high latitude, cold-water fishes because CTMin data are often censored at 0°C, even though eggs of some species can successfully develop at near freezing temperatures (Beitinger and Bennett, 2000; Beitinger *et al.*, 2000). Anthropogenic manipulations of water temperatures can expose fish to stressfully low and high temperatures, where performance or survival may be compromised (Caissie, 2006). Water project operations (e.g. dams and water diversions) can alter natural thermal regimes in green sturgeon habitat (Cloern and Jassby, 2012). For example, cold water releases from thermally stratified dams can abruptly reduce water temperatures by 10–15°C (Pike *et al.*, 2013), and temperatures are predicted to be altered even further under global climate change, with predictions of overall warmer river temperatures and intensified drought conditions (Cloern *et al.*, 2011).

Research has shown temperature to have pervasive effects on green sturgeon physiology (Mayfield and Cech, 2004), but temperature sensitivity differs depending on life-history stage, as seen in other fishes (Komoroske *et al.*, 2014). Further, fishes generally do not experience thermal stress passively and compensatory responses, such as the upregulation of Hsp and seasonal acclimatization can alleviate thermal stress (Prosser, 1991; Lindquist and Craig, 1988; Fangue *et al.*, 2011; Dalvi *et al.*, 2017). A life-history stage-specific overview of studies that examined the effects of temperature on green sturgeon is provided below.

#### Embryos

Temperatures experienced during fish embryogenesis can shift developmental trajectories, influence recruitment rates and have persistent effects on subsequent life-history stages (Martell and Kieffer, 2007; Schnurr *et al.*, 2014). Experimental assessments of the effects of water temperature on embryo development in green sturgeon are lacking and only a single study has been published. Van Eenennaam *et al.* (2005) assessed the effects of constant incubation temperatures, ranging from 11°C to 26°C, on embryo survival and development in three progenies. Embryos were obtained using artificial tank spawning with three mature females and five males, captured in the lower Klamath River by the Yurok Tribe (Van Eenennaam *et al.*, 2001). Hatching success was greatest between 14°C and 20°C, but the incidence of deformed embryos increased at temperatures ≥17.5°C. Elevated temperatures were particularly severe, and 100% mortality was observed at 23–26°C, although upper temperature tolerance differed slightly between progenies. The lower thermal tolerance limit was not identified, but hatching success was reduced at 11°C and hatched embryos were shorter. These findings suggest that 14–16°C is the optimal thermal range for successful development of green sturgeon because temperatures outside this range are associated with deleterious effects (i.e. reduced hatching success, higher deformity rates and smaller body sizes), but this needs to be replicated with more progenies and from females at different times of the spawning run, as maternal thermal history may influence progeny thermal tolerance (Le Roy *et al.*, 2017). In field studies, embryos at early stages of development have been collected using egg mats in the upper Sacramento River at 13.9°C (Brown, 2007) and between 11.3°C and 15.8°C (Poytress *et al.*, 2015), and in the Feather River between 16°C and 17°C (Seesholtz *et al.*, 2015). These observations fall within the broad range indicated by laboratory studies, although they suggest that spawning sturgeon may be required to accept less than optimal temperatures based on the available environmental conditions. No studies to date have examined temperature influenced differences in development in wild embryos.

Further research is required to understand the mechanisms underlying the emergence of embryonic abnormalities and mortality at unfavourable temperatures. Stage-specific assessments of abnormalities (e.g. at cleavage and neurulation) may provide insight into which developmental processes are most affected by suboptimal temperatures. For example, cleavage is one of the most heat-sensitive stages during embryogenesis in actinopterygian fishes, likely due to an exhaustion of maternally supplied Hsps (Motani and Wainwright, 2015). Indeed, abnormal cleavage in beluga sturgeon (*Huso huso*) has been observed at elevated temperatures (Dettlaff *et al.*, 1993). Embryo thermal tolerance is also modulated by egg quality, which is underscored by several factors, including stress exposure (e.g. hypoxia and contaminants), final ovarian stage of maturity, post-ovulatory ageing and egg handling (Bobe and Labbé, 2010). Green sturgeon fecundity is lower than white sturgeon, as they have much larger egg size, and consistent low to moderate fertilization rates have been observed in green sturgeon eggs over several years of artificial spawning induction (Van Eenennaam *et al.*, 2001, 2005, 2008, 2012). Of 23 females that ovulated in these studies, only four (17% of females) had eggs with fertility rates >80%, in comparison to most (~75%) white sturgeon, which routinely have >80% fertility rates (Van Eenennaam *et al.*, 2004). More research is required to characterize the thermal requirements of green sturgeon during embryogenesis and understand how additional stressors (e.g. hypoxia, contaminants and salinity) interact with thermal stress to affect egg quality and subsequent embryo survival.

#### Larvae

The impact of thermal stress on larval survival, growth and development has been the focus of several investigations. Elevated temperatures (>18°C) are particularly threatening to newly hatched larvae. Linares-Casenave *et al.* (2013) found survival of newly hatched larvae (hatch to yolk-sac depletion, Stage 45) to significantly decline between 22°C and 26°C and 100% mortality was observed at 28°C. An increase in notochord deformities (kyphosis) occurred at sub-lethal temperatures (20–26°C), and deformed larvae exhibited spiral swimming, which would likely compromise movements critical for predator avoidance and microhabitat selection. Thermal tolerance differed slightly between progenies, and heightened thermal tolerance was associated with an overexpression of Hsp60 and Hsp90.

Werner *et al.* (2007) conducted a similar study, where the capacity of newly hatched larvae to tolerate and recover from acute thermal stress was assessed. Larvae were exposed to one of three temperature treatments until yolk-sac absorption: constant 17°C (control), exposed to 26°C for 3 days and returned to 17°C or constant 26°C. Overall, deformity rates were lowest in larvae continuously maintained at 17°C and highest in larvae continuously exposed to 26°C. In the recovery treatment, ~33% of larvae exhibited deformities after 3 days of exposure to 26°C, but this rate halved once larvae were returned to cooler water, demonstrating some capacity to recover from thermal spikes. Deformed larvae also exhibited modified Hsp expression, where Hsp60 levels were lower but Hsp72 and Hsp78 levels were higher compared to healthy larvae.

Post yolk-sac larvae appear more resilient to elevated temperatures than newly hatched larvae. Allen *et al.* (2006b) assessed the effects of elevated and cycling thermal regimes (control 19°C; 19–24°C and 24°C) on growth rates in post yolk-sac larvae (15 days post-hatch, dph). Elevated and cycling thermal regimes had no adverse effects on growth rates, and growth was highest in fish maintained at 24°C. Accelerated growth rates at 24°C were facilitated by increased food consumption, but it was noted that growth of wild larvae could be limited at elevated temperatures if prey is not abundant.

There are few reported observations of larval green sturgeon in field studies; however, some yolk-sac larvae have been collected in the upper Sacramento River above Bend Bridge (Rkm 415) at temperatures of 13.2°C (Brown, 2007) and 12.9 ± 1.0°C (Poytress *et al.*, 2015). While these temperatures are substantially lower than those at which developmental abnormalities have been observed in lab studies, it should be noted that water temperatures in this reach are held at <13.3°C by Order 90-5 of the California State Water Resources Control Board for the benefit of winter-run Chinook salmon. It remains unclear what temperature larval green sturgeon seek out or prefer in natural field conditions.

#### Juveniles

More is known regarding the thermal requirements of juvenile green sturgeon compared to other life-history stages. A comprehensive study by Mayfield and Cech (2004) evaluated the thermal sensitivity of food consumption and conversion efficiency, growth rates, oxygen consumption, ventilatory frequency, volitional activity, thermal preference and swimming performance in juveniles. These data were collated to provide a temperature target for optimal bioenergetic performance. Bioenergetic performance was estimated to be optimal between 15°C and 19°C, as energetic demands increased beyond sustainable limits at higher temperatures. Swimming performance was compromised at 24°C in age-1 juveniles, suggesting that unseasonably high temperatures may impede critical movements, such as outmigration, predator avoidance and habitat selection. However, younger juveniles (155 dph) may be less vulnerable to high temperatures, as Allen *et al.* (2006a) found swimming performance to be greater at 24°C compared to 19°C. Young juveniles (47 dph) have also been shown to respond to daily thermal fluctuations, with fish exposed to wide temperature fluctuations (11–21°C day^−1^) able to maintain swimming performance across a wider range of temperatures and having improved growth compared to fish maintained at stable temperatures (Rodgers *et al.*, 2018).

Hsp responses have been elicited in juvenile green sturgeon by both acute and chronic exposures to high temperatures (Allen *et al.*, 2006a; Wang *et al.*, 2013). Mucus appears to be the most sensitive tissue to Hsp70 induction and white muscle is the least responsive (Wang *et al.*, 2013). Green sturgeon exhibited significantly lower Hsp70 responses compared to white sturgeon, suggesting green sturgeon may be more vulnerable to the damaging effects of elevated temperatures. Temperature can also modulate the stress response in juvenile sturgeon. Lankford *et al.* (2003) assessed the physiological stress response (i.e. plasma cortisol and lactate concentrations) of age-0 fish exposed to a 1-minute air emersion at 19°C (control temperature) and 11°C. The magnitude of the stress responses did not differ between temperatures, but recovery was significantly prolonged at 11°C, providing evidence that juvenile sturgeon may therefore be more susceptible when recovering from stress at cold temperatures.

#### Sub-adults, adults and spawning adults

Very little is known about the thermal requirements of adult green sturgeon. During the peak Klamath River spawning migration (April–May) river temperatures generally range over 8–15°C under normal precipitation years (Van Eenennaam *et al.*, 2006). Spawning migrations begin earlier and are truncated during dry years when water temperatures are elevated and flows are reduced (Van Eenennaam *et al.*, 2006). Ripe northern DPS adults have also been reported in the Rogue River or when water temperatures ranged between 9.3°C and 18°C (Erickson and Webb, 2007). Similarly, spawning of the southern DPS in the Sacramento River occurred at temperatures ranging between 9.6°C and 17.6°C, with a daily mean ± SD of 13.5 ± 1.0°C, and temperatures ranged between 16°C and 17°C during spawning in the Feather River (Poytress *et al.*, 2015; Seesholtz *et al.*, 2015). Additionally, when sub-adults were manually tracked in the San Francisco Estuary, they selected temperatures ranging from 14.5°C to 20.8°C as measured in the water column at the depths at which they were swimming (Kelly *et al.*, 2007). Adult green sturgeon have been tracked along the coast of Oregon and in Willapa Bay and at temperatures ranging from 9.5°C to 21.9°C (Huff *et al.*, 2011; Moser and Lindley, 2007).

Remote recordings of water/body temperature of free-ranging adult green sturgeon would provide valuable thermal preference data. An implantable multi-channel biotelemetry system for measuring blood flow and body temperature has been tested in sub-adult green sturgeon (age 3; Grans *et al.*, 2009). This system allowed the calculations of cardiac output as a function of heart rate and stroke volume during an acute thermal challenge. Temperatures were increased from 19°C to 26°C, and a moderate increase in cardiac output was observed as temperature increased. This methodological advance offers the possibility of collecting important physiological data on free-ranging adults, and interactions between thermal preference and sex, age, body size or reproductive status may be identified.

#### Management actions and data gaps

**Figure 3:**
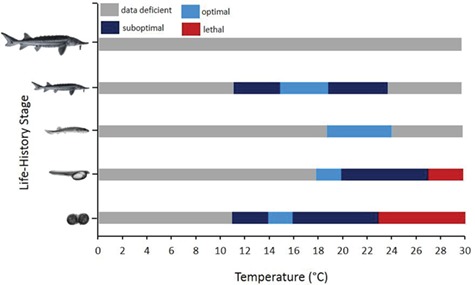
Life-history stage specific thermal ranges of green sturgeon (*Acipenser medirostris*) where survival and performance (growth, swimming performance and normal development) are optimal (light blue) and suboptimal (dark blue). Lethal temperatures are red and grey bars represent D.D. temperatures. Life-history stages are represented in order as embryos, yolk-sac larvae, post yolk-sac larvae, juveniles and adults. Data were extracted from peer-reviewed publications (*SI*) on the northern DPS on full feed rations.

Temperature ranges where physiological performance is optimal differs between life-history stages (Fig. 3) and management actions should be geared towards the most vulnerable stage. Early developmental stages (embryos–yolk-sac larvae) are highly sensitive to thermal perturbations and only narrow thermal windows (embryos, 14–16°C; yolk-sac larvae, 16–18°C) ensure survival and growth are optimized and deformities are minimized (Fig. 3). Physiological performance is maintained across a wider breadth of temperatures in post yolk-sac larvae and juveniles, but the thermal requirements of adults remain largely uncharacterized (Fig. 3). The overall mean effect of elevated temperatures (>19°C) on green sturgeon physiology are shown in Fig. 2A. Growth and embryo hatching success are significantly reduced at elevated temperatures, likely owing to metabolic demands exceeding energy supply (Fig. 2A). The incidence of deformities increases at elevated temperatures, along with increased expression of Hsps (Fig. 2A). Insufficient data exist on the thermal sensitivity of swimming performance, despite this trait representing an important fitness correlate (Plaut, 2001).

Ensuring water temperatures are maintained within optimal ranges during peak spawning periods (April–May) in critical rearing habitat is a potential management strategy. Maintaining water temperatures below 20°C may benefit green sturgeon, particularly during early development (Fig. 2A, Fig. 3); however, more data are required before specific thermal ranges can be recommended. An understanding of how cold temperatures (≤11°C) influence green sturgeon physiology is lacking across all life-history stages (Fig. 3); however, a recent study evaluating the effects of temperature on growth performance found growth to cease at 11°C suggesting that temperatures at or below this point will likely result in time limited survival (Poletto *et al.*, 2018). Hypolimnetic water releases from dams within the Sacramento–San Joaquin (S-SJ) watershed can expose green sturgeon to unseasonably low temperatures during critical spawning periods (Pike *et al.*, 2013). Research efforts should aim to identify lower temperature limits for survival, growth and swimming performance across all life stages.

Thermal requirements for embryos and adults are poorly characterized and represent fruitful research avenues. Specifically, additional work is required to understand transgenerational effects on thermal tolerance (Rossiter, 1996). Temperatures experienced by parental fish can influence offspring phenotype and may facilitate transgenerational plasticity (Mousseau and Fox, 1998; Burt *et al.*, 2011). Progeny of spawners from an early spawning migration of starry sturgeon (*A. stellatus*) had a narrower range of temperature tolerance than progeny of spawners from the late migration whose parents experienced warmer waters during migration (Sytina and Shagaeva, 1987). These authors reported an upward shift in the temperature limits of lethal, sublethal and suboptimal effects by 2–3°C in *A. stellatus* embryos. Moreover, inter-progeny variation in thermal tolerance of green sturgeon reported (Van Eenennaam *et al.*, 2005; Linares-Casenave *et al.*, 2013) may partially reflect transgenerational plasticity (Rossiter, 1996; Mousseau and Fox, 1998). Lastly, future experiments should be designed to maximize ecological relevance by mimicking natural thermal regimes and exposing fish to multiple heterologous stressors in unison. Almost all studies (c.f. Allen *et al.*, 2006b; Rodgers *et al.*, 2018) used constant temperatures, but natural river temperatures with daily fluctuations should be mimicked.

### Salinity

Green sturgeon are truly anadromous and spend a large portion of their life in the ocean (Allen and Cech, 2007). Mature adults undertake long-distance, seasonal migrations between marine and freshwater habitats to spawn (Lindley *et al.*, 2008). Eggs and larvae develop in freshwater and juveniles may remain in freshwater habitats for up to 3 years prior to outward migration (Nakamoto *et al.*, 1995). Juvenile sturgeon undergo physiological remodelling in preparation for seaward migrations (Allen *et al.*, 2009, 2011). The timing of this physiological remodelling is age and size dependent and occurs at a young age relative to other sturgeons (Allen *et al.*, 2009). Exposure to salinity prior to physiological remodelling can disrupt osmotic homeostasis (e.g. plasma osmolality and muscle moisture; Fig. 2B) and increase energetic costs through the upregulation of ionoregulatory mechanisms (Boeuf and Payan, 2001). Salinity levels within the San Francisco Bay Delta (SFBD) are forecasted to increase under climate change due to sea-level rise, seawater intrusion and altered precipitation patterns (Cayan *et al.*, 2008). Therefore, recent research efforts have focused on understanding the timing of physiological remodelling, behavioural responses to salinity gradients and the costs associated with salinity exposure in green sturgeon. Data on the effects of elevated salinity on early life stages (embryonic and larval stages) are currently unavailable.

#### Juveniles

Research has been geared towards understanding the timing of physiological adjustments associated with seawater acclimation in juveniles. Findings have shown that both age and body size modulate the timing of seawater acclimation (Allen and Cech, 2007). Short-term exposure (72 h) to elevated salinity (20–33 ppt) during early life (<100 dph) causes profound declines in survival (Allen *et al.*, 2011). However, juveniles can tolerate brackish (10 ppt) to full seawater (33 ppt) when more developed (i.e. 170 dph, ~15 g) but at the cost of reduced growth (Allen and Cech, 2007; Allen *et al.*, 2011) resulting from the disruption of ionic homeostasis (increased plasma osmolality and muscle moisture; Fig 2B). At 1.5 years of age (1.5 kg) juveniles show complete seawater acclimation with no costs evident (Allen and Cech, 2007; Sardella and Kültz, 2009). Juvenile green sturgeon ranging in size from 19 to 58 cm TL were captured by gill net and otter trawl in the freshwater region of the Sacramento–San Joaquin Delta near Sherman Island (Radtke, 1966). It remains unclear at what point in development this species moves into saline waters in the wild, but sub-adult fish of ~1 m TL were captured and tracked in the San Francisco Bay Estuary in water ranging from 8.8 to 32.1 ppt (Kelly *et al.*, 2007).

Physiological changes underlying seawater acclimation have been characterized and include increased gill and pyloric ceca sodium potassium-ATPase (NKA) activity, gill mitochondria-rich cell size, lamellar length, body condition, hematocrit and thyroid hormone levels (T_3_ and T_4_) (Allen *et al.*, 2009, 2011; Haller *et al.*, 2015). Behavioural responses to salinity have also been examined in juveniles, finding that at 196 dph juveniles display a preference for seawater regardless of acclimation salinity (Poletto *et al.*, 2013). These findings are consistent with the timing of the development of seawater tolerance and suggest juveniles actively seek out saline environments at this age.

Salinity tolerance can be modulated by the presence of additional stressors. Haller *et al.* (2015) found salinity tolerance to be significantly impaired when food was restricted in 240 dph juvenile sturgeon. Food-restricted fish showed altered hematological indexes, plasma parameters (osmolality, glucose, lactate and cortisol), and gill and pyloric ceca enzymatic activity. Similarly, Vaz *et al.* (2015) found combined exposure to seawater (16 and 32 ppt) and food restriction synergistically decreased body condition factor. Upper thermal tolerance (CTMax) can also change in response to environmental salinity (Sardella *et al.*, 2008). For example, CTMax increased 0.5°C in juveniles (58.4 ± 2.5 g) acclimated to brackish water (10 ppt) compared to juveniles acclimated to freshwater or bay water (24 ppt), suggesting energy demands were lowest in brackish, near-isosmotic water.

#### Management actions and data gaps

Although it is appreciated that salinity in the SFBD is projected to increase under climate change, very little is known regarding the effects of salinity exposure on early life-history stages. Elevated salinity will likely impair hatching success and growth and development of larvae, but this has yet to be explored. Nonetheless, it is evident that young juveniles (<100 dph) experience heightened mortality rates in saline waters (Allen *et al.*, 2011) and salt intrusion should be mitigated in nursery habitats. Salinity can be maintained at low levels by manipulating dam releases to increase river flows, termed ‘flushing’ (Liu and Liu, 2014). Moreover, climate change scenarios predict salinity levels to rise in tandem with water temperatures (Cayan *et al.*, 2008), along with changes in other environmental variables (e.g. aquatic pH). Therefore, an understanding of the interactions among elevated temperatures, salinity, pH and other environmental stressors on the physiology of early life-history stages is essential for future-focused management plans.

### Food restriction

Low food availability is a pervasive stressor in developed waterways, and record declines in zooplankton biomass have been observed in the SFBD over the past four decades (Cloern *et al.*, 1983; Carlton *et al.*, 1990; Cloern *et al.*, 2014; Winder and Jassby, 2011). Diminished food resources stem from exotic species, namely the overbite clam (*Corbula amurensis*), and flow regulation modifying food-web structure and lowering primary productivity (Cloern *et al.*, 1983; Cloern and Jassby, 2012). Food scarcity can disrupt the energy budget of fish and lower stress tolerance via a divestment of energy towards protective molecular and cellular responses (Sokolova, 2013). Lower stress tolerance decreases fish resilience to additional stressors such as elevated temperatures or salinity. However, the most well-documented effect of food restriction is a decrease in somatic growth (Jobling, 1994). Slowed growth rates, particularly during early life stages, can have important consequences because smaller body sizes often confer increased mortality rates (Carlson *et al.*, 2008) and reduced competitiveness over resources (Johnsson *et al.*, 1999). The effect of food restriction on growth, development and susceptibility to additional stressors have been examined in green sturgeon, although studies are skewed towards juveniles and very little is known about food preferences in the wild at any life stage.

#### Larvae

Green sturgeon larvae typically initiate exogenous feeding at 10–16 dph, but water temperabuture modulates yolk-sac absorption rates and time to exogenous feeding (Van Eenennaam *et al.*, 2001). The digestive tract of sturgeon develops during the larval stage and is sensitive to changes in food availability (Gisbert and Doroshov, 2003). Gisbert and Doroshov (2003) found food deprivation in green sturgeon larvae (hatching—31 dph) to cause pathological changes after 5 days and significant deterioration of the digestive system after 10–15 days, evident as tissue degeneration, necrosis and shrinkage of digestive epithelia. These findings suggest there is a critical period of 4–5 days following hatching where larvae must feed to ensure the healthy development of their digestive system.

#### Juveniles and adults

Food restriction can impact growth rates, body condition, energy stores and stress tolerance in juvenile green sturgeon. Intuitively, food restriction decreases growth rates (Fig. 2C), feeding efficiency, body condition as well as whole-body lipid and protein stores (Haller *et al.*, 2015; Zheng *et al.*, 2015; Vaz *et al.*, 2015; Verhille *et al.*, 2015; Poletto *et al.*, 2018), but effects on stress tolerance are less uniform. Haller *et al.* (2015) found that food restriction impaired osmoregulation capacity in juveniles (222 dph), and exposing food-restricted fish to full-strength sea water (32 ppt) resulted in high mortality rates within 72 h. Other studies have investigated the effect of food restriction on heat tolerance and Hsp expression (Wang *et al.*, 2013; Zheng *et al.*, 2015; Lee *et al.*, 2016; Verhille *et al.*, 2015). Findings have been mixed, but overall food restriction does not significantly affect upper thermal limits (CTMax) or the expression of Hsps (Fig. 2C).

#### Management actions and data gaps

Overall, food restriction is known to have irreversible impacts on the development of the digestive tract in young larvae and affect growth rates and energy stores in juveniles and compensatory growth following periods of food restriction were not seen in a recent study (Poletto *et al.*, 2018). Restoration of natural food-web dynamics may alleviate the threat of food scarcity. Reversing disrupted food-web linkages in the SFBD may be achieved by restoring natural water flow regimes and controlling invasive primary consumers that disrupt the food-web from its base (Cloern *et al.*, 1983; Strayer, 2010). Research on the effects of food restriction in green sturgeon and other fishes is still in its infancy. No empirical data exist on the effect of food restriction on green sturgeon adult body condition, but bioenergetic models suggest spawning frequency may be reduced under low prey availability scenarios (Borin *et al.*, 2017). It is recommended that future research efforts explore the effects of food restriction across all life-history stages to further our understanding of the impacts of food restriction on a range of factors including disease susceptibility, contaminant uptake rates and reproductive investment. Dietary preferences of green sturgeon in the wild across all life stages should also be investigated in order to both identify critical habitat for management and understand the impacts of food restriction on bioenergetics.

### Contaminants

The SFBD is listed as an ‘impaired waterway’ under the US Clean Water Act because many contaminants exceed regulatory thresholds (SWRCB, 2010). Contaminants enter the SFBD from a variety on inputs, including municipal and industrial wastewater, urban and agricultural runoff, and direct pesticide application (Fong *et al.*, 2016). Contaminants associated with this listing include heavy metals (e.g. mercury, cadmium, copper and zinc), pesticides (e.g. chlordane and organophosphate insecticides) and chlorinated compounds (e.g. polychlorinated biphenyls and dioxins) (Fong *et al.*, 2016). Recent studies by Moschet *et al.* (2014) targeting pesticides and pharmaceuticals detected 17 common compounds across 51 samples, highlighting risks of potential synergism resulting from exposure to a multitude of co-occurring contaminants. Uptake of contaminants in fishes can occur through aqueous exposure (e.g. branchial exposure during gill ventilation) and dietary exposure (consuming contaminated prey) (Arnot and Gobas, 2006). Contaminant exposure can disrupt organismal functions across all levels of biological organization, from cellular mechanisms to whole-animal performance, impacting reproduction and population dynamics. Research investigating the effects of contaminants on green sturgeon is limited to a small number of studies examining the effects of exposure to selenium, carbaryl and methylmercury, a legacy contaminant in the SFBD. Selenium is an essential micronutrient to all vertebrates but is toxic at higher concentrations and aquatic organisms are particularly vulnerable (Janz *et al.*, 2010). Exposure to elevated selenium levels has been shown to result in developmental abnormalities and skeletal deformities in multiple fish species (Holm *et al.*, 2005; Shi *et al.*, 2018; Kupsco and Schlenk, 2016). Agricultural runoff over seleniferous soils and water discharge from industrial operations are the main sources of excessive selenium inputs in the SFBD (Lemly, 2004). The SFBD also suffers from high levels of mercury (Hg) contamination, due to intense hydraulic gold mining operations from 1850 to 1960 (Dasmann, 1999). Once in the water, bacteria and fungi convert inorganic Hg into methylmercury (MeHg), a highly toxic form, owing to its high bioavailability and affinity to bind to thiol groups (Harris *et al.*, 2007). Exposure and responses to numerous contaminant classes vary depending on life-history stage (Brooks *et al.*, 2012), as well as with physicochemical properties such as temperature (Maulvault *et al.*, 2016) and salinity (Smylie *et al.*, 2016), and management actions need to be tailored accordingly. The primary studies evaluating the effect of contaminants on sturgeon address MeHg and selenomethionine (SeMet). There is a lack of information regarding the effects posed by a multitude of contaminants detected in the SFBD.

#### Embryos

Early life-history stages are generally most susceptible to the detrimental effects of contaminants (McKim, 1977; Foekema *et al.*, 2012). Embryos can be exposed to contaminants through direct exposure to water and contact with sediment or via maternal transfer (Niimi, 1983). Green sturgeon embryogenesis occurs during a period of direct contact with sediment, which is potentially laden with multiple contaminants. Sediments act as pollutant sinks and release contaminants back into the water column following disruption or bacterial activity, thus the sediment–water interface is an area of highly concentrated pollution. Despite green sturgeon embryos being at a high risk of contaminant exposure, no studies have assessed the effects of contaminant exposure in this life stage. In other fishes, exposure to contaminants during embryogenesis has led to increased mortality, slowed development, increased heart rates and dysfunctional lateral lines (Johnson *et al.*, 2007; Vardy *et al.*, 2013). Chorionic permeability is known to differ greatly between fish species and does not necessarily provide sufficient protection from anthropogenic contaminants. Studies conducted by Weis and Weis (1989) have demonstrated differences in adsorption of methylmercury by the chorion vs. developing embryos, correlating with population tolerance in killifish, (*Fundulus heteroclitus*). Green sturgeon embryos may also be exposed to maternally transferred contaminants. For example, studies have reported selenium concentrations in ovaries and ovulated eggs sampled from white sturgeon in the SFBD at levels that far exceed reproductive toxicity thresholds (Kroll and Doroshov, 1991; Lemly, 1996; Linares-Casenave *et al.*, 2015). Several reviews and expert panels have highlighted the need to conduct studies on the effects of multiple contaminants on green sturgeon embryos (Johnson *et al.*, 2010; Brooks *et al.*, 2012; IEP MAST, 2015; Fong *et al.*, 2016), as exposure may contribute to recruitment failure.

#### Larvae

Sturgeon yolk-sac larvae take refuge in sediments prior to the onset of exogenous feedings and are likely to be chronically exposed to high concentrations of contaminants (Richmond and Kynard, 1995; McAdam, 2011). Many chemicals are lipophilic and thus will adsorb to the yolk-sac and be dispersed throughout the developing larvae. Yolk-sac larvae may be particularly vulnerable to contaminant exposure because they are energy limited (yolk-sac dependent) and energy may be redirected from growth and organogenesis towards enzymatic detoxification of contaminants, repair mechanisms and toxicant elimination (Brooks *et al.*, 2012). Once exogenous feeding initiates, larvae spend less time in the sediment but contend with dietary intake of contaminants through prey consumption. Very little is known regarding the effects of contaminants on green sturgeon larvae, and only a single study on post yolk-sac larvae has been published. Silvestre *et al.* (2010) examined the effects of selenium exposure on survival, morphology and protein expression profiles in green and white sturgeon. Selenium stress (body burden of 8 μg g^−1^) induced developmental abnormalities (i.e. peritoneal and pericardial oedemas and spinal deformities), altered behaviour (i.e. lethargy) and physiological changes (i.e. bradycardia and internal haemorrhaging). Overall, mortality rates and mechanistic responses, determined through proteome evaluations, in selenium-exposed fish were higher in green compared to white sturgeon. These findings suggest green sturgeon exhibit greater sensitivity to contaminant exposure; although it should be noted that additional research is needed as Silvestre *et al.* (2010) only tested one family exposed to a single contaminant.

#### Juveniles

More is known regarding contaminant exposure in juvenile green sturgeon compared to other life-history stages. Studies have focused on the effects of dietary exposure of l-SeMet and methylmercury (MeHg) on growth, tissue burden and histopathology (Lee *et al.*, 2011, 2012; De Riu *et al.*, 2014). De Riu *et al.* (2014) exposed juvenile green and white sturgeon to four levels of dietary SeMet (0, 50, 100 or 200 mg SeMet kg^−1^) for 8 weeks. SeMet diets were associated with reduced body lipids, reduced growth, kidney and liver abnormalities and increased mortality rates in a dose-dependent pattern. Of concern, even the low SeMet diet, which closely reflected concentrations found in prey items of juvenile sturgeon (benthic macro-invertebrates), caused detrimental effects. Overall, the adverse effects of dietary SeMet were more pronounced in green compared to white sturgeon, providing another line of evidence suggesting green sturgeon are more susceptible to contaminants. In a similar study, juvenile green sturgeon were exposed to one of four dietary concentrations of MeHg (0, 25, 50 and 100 mg MeHg kg^−1^) for 8 weeks (Lee *et al.*, 2011, 2012). Methylmercury burden was detected in all assessed tissues, and highest concentrations were detected in the kidney. Green sturgeon had a higher MeHg tissue burden in the liver relative to white sturgeon, but tissue burden was higher in all other white sturgeon tissues. Despite this, dietary MeHg exposure had greater negative effects on survival, growth and development in green sturgeon compared to white sturgeon. The disparity in responses to dietary contaminants in green and white sturgeon suggest that the two species have different functional or structural capabilities in dealing with contaminant (e.g. uptake, detoxification and elimination capacities) and show that white sturgeon is an inappropriate surrogate species for green sturgeon (Lee *et al.*, 2011, 2012). There are no additional toxicity data for green sturgeon, beyond the limited MeHg and SeMet studies detailed above.

#### Sub-adults and adults

Sub-adult and adult life stages of green sturgeon may be less exposed to contaminants because the marine environment is more distant from pollutant sources than freshwater systems (Vörösmarty *et al.*, 2010). However, adults forage in polluted habitats during obligate migratory movements (Moser and Lindley, 2007) and the longevity of green sturgeon makes adults vulnerable to bioaccumulation and biomagnification of toxins. Indeed, Se tissue burden appears to increase with age in white sturgeon, suggesting significant bioaccumulation occurs prior to reaching sexual maturity (Linares-Casenave *et al.*, 2015). Stomach content analyses show that adult green sturgeon feed on two species of burrowing shrimp (*Neotrypaea californiensis* and *Upogebia pugettensis*) in Willapa Bay and Grays Harbor (WA, USA)—major areas of commercial oyster cultivation (Dumbauld *et al.*, 2008). Burrowing shrimp are considered a pest because they impair oyster growth and survival, and widespread applications of imidacloprid pesticides have occurred to control shrimp abundance. Concerns that green sturgeon are exposed to imidacloprid via shrimp consumption and interactions with sediment porewater prompted Frew *et al.* (2015) to conduct a study to characterize imidacloprid exposure. Sturgeon feeding pits (shallow depressions) were counted before, during and after imidacloprid applications at 24 field sites and compared to reference sites where no imidacloprid applications occurred. Results suggested that green sturgeon preferentially fed on imidacloprid-impaired shrimp as the number of feeding pits following a pesticide application was 78% higher compared to reference sites. Total imidacloprid uptake, from both branchial and dietary intake, was also modelled and it was estimated that porewater exposure contributes more than shrimp consumption (Frew *et al.*, 2015). Concentrations and durations of imidacloprid exposure were suggested to be lower than toxic thresholds; however, these estimates were based on toxicity assessments in white sturgeon (Frew and Grue, 2015) and several studies have demonstrated the unsuitability of white sturgeon as a surrogate species (Silvestre *et al.*, 2010; Lee *et al.*, 2011, 2012; De Riu *et al.*, 2014). Similarly, the effect of another pesticide used to control burrowing shrimp, carbaryl, has been studied in white sturgeon as a surrogate species (Troiano and Grue, 2016) but this work provides little information on the vulnerability of green sturgeon.

#### Management actions and data gaps

Overall, certain contaminant classes appear to accumulate in green sturgeon tissues through chronic exposure, leading to significant increases in body burden and mortality (Fig. 2D). At sub-lethal levels, contaminants lower green sturgeon growth rates (Fig. 2D), potentially reflecting a disruption to energy homeostasis. Contaminant exposure may cause fish to divest energy into fitness-related activities, such as growth, predator avoidance and reproduction, to mitigate damage (Goto and Wallace, 2010). Gearing future research efforts to understand effect-based, sub-lethal physiological costs (e.g. impaired swimming performance or elevated maintenance costs) of contaminant exposure may effectively assess proximate causes of green sturgeon declines (Brooks *et al.*, 2012). Data are lacking across all life-history stages, particularly during critical early life periods when contaminant sensitivity may be heightened (McKim, 1977). Measuring the effects of contaminant exposure along with other stressors (e.g. elevated temperatures, salinity and turbidity) or contaminant mixtures would better reflect ecologically relevant conditions. Additional stressors may influence contaminant uptake, accumulation, excretion and toxicity (Sokolova and Lannig, 2008). Once a greater understanding of green sturgeon susceptibility to a multitude of contaminant classes present in the SFBD is gained, beyond that of MeHg and SeMet, a life-history stage-specific management plan can be put into place, which explicitly considers temporal and spatial changes in habitat use and exposure to additional stressors.

### Movement barriers

Green sturgeon declines have been linked to extensive fragmentation of spawning and rearing habitat, particularly in the S-SJ watershed (Cloern and Jassby, 2012). The S-SJ watershed has been extensively altered since the mid-1800s when hydraulic gold mining operations were pervasive and exponential human population growth followed (Dasmann, 1999). The S-SJ watershed currently supplies water to ~1 million hectares of agricultural land and over 25 million people (Service, 2007). To meet this demand and provide flood protection, tens of water reservoirs and over 3300 water diversion pipes have been constructed within the river system (Herren and Kawaski, 2001; Cloern and Jassby, 2012). These alterations are part of the two largest water diversion operations worldwide (State Water Project and the Central Valley Project).

Waterway infrastructure can impede fish movements by creating physical (e.g. dams), hydraulic (e.g. excessive water velocities), physiochemical (e.g. low dissolved oxygen) and behavioural (e.g. low light levels) barriers. The survival and reproductive success of green sturgeon and other fishes are tied to their capacity to move freely and efficiently within the environment at all life stages (Fausch *et al.*, 2002). Small-scale movements are vital for foraging and predator avoidance, whereas large-scale movements are necessary for reaching spawning grounds, juvenile outmigration, habitat selection and maintaining population connectivity (Clapp *et al.*, 1990; Gowan and Fausch, 2002; Yamamoto *et al.*, 2004). Successful navigation of instream barriers is related to fish swimming capabilities and behavioural responses to water flow (Adams *et al.*, 2007; Boysen and Hoover, 2009). To this end, research efforts have characterized green sturgeon swimming capacity and coupled this with behavioural responses to water flow and instream structures. Moreover, the efficacy of retrofitting water diversion pipes with a range of deterrents and fish-protection devices has been assessed (Poletto *et al.*, 2014b; Poletto *et al.*, 2015). Together these findings can guide conservation efforts aimed at providing unimpeded passage of green sturgeon.

#### Water diversions

Water diversion pipes are designed to maximize hydraulic capacity and water extraction efficiency. Diversion velocities far exceed the swimming capacities of many fishes, and many are consequently drawn inside (i.e. entrained; Grimaldo *et al.*, 2009). Entrained fishes can be physically damaged by pump operations or left stranded in irrigation fields (Baumgartner *et al.*, 2009). Juvenile green sturgeon may spend up to 1.5 y within the S-SJ watershed and likely encounter multiple water diversion pipes during daily movements and outmigration. Mussen *et al.* (2014) were the first to assess the susceptibility of juvenile green sturgeon (~35 cm FL) to entrainment in water diversion structures. Experiments were held in a large river-scale simulation flume and diversion flows were set to replicate field conditions. Entrainment rates were high, and fish were rapidly entrained (<1 s) after passing the diversion pipe. Extrapolations of findings predicted that ~22% of outmigrating juveniles could become entrained if they passed by a single water diversion and this number increased markedly with the number of diversions passed. However, entrainment rates were reduced dramatically by lowering water extraction velocities (from 0.57 to 0.28 m^3^ s^−1^), suggesting that limiting inflow velocities may be a successful management strategy.

Building on this, Verhille *et al.* (2014) characterized the swimming capacity of larval and juvenile green sturgeon by measuring critical swimming speeds (*U*_crit_). Absolute swimming speeds increased with fish size and age, likely due to greater muscle mass and enhanced energetic efficiency (Beamish, 1978; Videler, 1993). However, 50 cm-long (TL) juveniles were exceptions to this trend and exhibited highly variable and lower-than-predicted swimming speeds. Verhille *et al.* (2014) noted that juveniles of this size typically develop salt-water tolerance in preparation for outmigration, and this decline in swimming performance likely represented an energetic trade-off between physiological remodelling required for seawater acclimation and locomotor performance. Indeed, this finding has been corroborated with another study, where a decline in *U*_crit_ corresponded with the onset of hypersaline osmoregulatory capacities in similarly sized juveniles (Allen *et al.*, 2006a).

Ontogenetic changes in swimming performance highlight the need to specify limits on water diversion inflow velocities according to season and location within the S-SJ watershed. Entrainment risk is thought to be greatest at nighttime as larval and juvenile green sturgeon perform nocturnal foraging and migration movements (Kynard *et al.*, 2005). Therefore, Verhille *et al.* (2014) suggested water diversion inflow limits only be enforced at nighttime. Larval fish were identified as the weakest swimmers and it was recommended that inflow velocities, within the mid-reaches of the Sacramento River, be restricted to ≤0.29 m s^−1^ from May through summer, to match peak rearing times (Verhille *et al.*, 2014). Young juveniles (~55 dph; TL: 7 cm) were the strongest swimmers and it was suggested that nighttime water diversion inflow velocities are limited to ≤ 0.54 m s^−1^ in the mid-reaches of the Sacramento River from July through May. Outmigrating juveniles (TL: 8–50 cm) were also identified to be susceptible to entrainment, owing to their weak swimming ability as they prepare for seawater entry. Therefore, it was recommended that nighttime diversion inflows are limited to ≤ 0.40 m s^−1^ in the middle and lower river reaches during peak movements (October–November). These data offer baseline guidelines that require further testing before implementation.

Taking a different approach, installing deterrents or fish-protection devices at water diversion pipes may lower fish entrainment rates without reducing hydraulic efficiency. The efficacy of two sensory deterrents, a strobe light and acoustic vibrations, in eliciting avoidance behaviour and minimizing entrainment has been assessed in juvenile green sturgeon (Poletto *et al.*, 2014a,b). Both sensory deterrents proved ineffective and had no effect on entrainment rates, despite reports of success in other species (Maes *et al.*, 2004; Hamel *et al.*, 2008). Poletto *et al.* (2014b, 2015) also evaluated the efficacy of several physical fish-protection devices in lowering entrainment rates. Retrofit designs included a modified upturned pipe configuration and the addition of a protective screen, louvre box, trash-rack box or perforated cylinder. All retrofit designs significantly reduced entrainment rates. For example, the addition of a protective screen lowered entrainment rates from 44% in an unmodified pipe to 13%. The upturned pipe configuration allowed water to be withdrawn from the middle of the water column rather than the bottom. This retrofit design was particularly effective at reducing entrainment rates, likely owing to the benthic behaviour of green sturgeon. However, Poletto *et al.* (2014a) question the feasibility of this design because it can only be installed in water bodies with sufficient depth to avoid navigation hazards by boats and could increase entrainment risk for pelagic species. Other retrofit designs (i.e. louvre box, trash-rack box and perforated cylinder) reduced entrainment risk by 60–96%. The effectiveness of these designs was postulated to be linked to physical protection, and inflow velocities being distributed across a greater area, enabling fish to detect flow changes from a greater distance and initiate avoidance behaviour.

Behaviour of juvenile green sturgeon near water diversions with protective screens has also been investigated. Although fish exclusion screens can reduce entrainment rates, fish can become impinged on screens (Poletto *et al.*, 2014b). Screen contacts can injure fish and cause heightened levels of physiological stress (e.g. acidosis from exhaustion; Young *et al.*, 2010). Poletto *et al.* (2014b) compared impingement rates between juvenile green and white sturgeon, finding that green sturgeon were impinged twice as often and spent more time near the screen. Impingement rates also varied according to fish size and age (Poletto *et al.*, 2018). A comparison between three size classes of green sturgeon (small juveniles, 9.2 cm FL; intermediate juveniles, 18.8 cm FL; and older juveniles, 29.5 cm FL) identified a general negative trend between fork length and the number of body contacts with the screen. Intermediate-sized juveniles (18–20 cm) were exceptions and contacted the screens more frequently than smaller fish. This size range was correlated with the time when osmoregulatory capacity is developed (Allen and Cech, 2007; Allen *et al.*, 2009), providing yet another line of evidence to suggest juveniles of this stage are particularly susceptible to water diversions.

#### Water reservoirs

Water impoundments (e.g. dams and weirs) can act as large-scale movement barriers and block access to critical spawning grounds (Bednarek, 2001). Adult green sturgeon migrate upstream to freshwater habitats to spawn and perform long-distance movements within freshwater reaches during the spawning season (Thomas *et al.*, 2014). Dams can impede upstream movements by creating physical blockades (e.g. closed gates) and hydraulic barriers in the form of excessive water velocities below flow-control gates (Brown, 2007). Fish ladders (i.e. fishways) have been installed at many dams to facilitate fish passage, but these structures are designed for salmonids with superior swimming abilities compared to green sturgeon (Mallen-Cooper and Brand, 2007).

To assess the effects of dams and altered flow regimes on the geographic distribution of green sturgeon, Mora *et al.* (2009) characterized habitat preference by correlating environmental characteristics (i.e. river discharge, water velocity, channel gradient and temperature) with over 2000 green sturgeon sightings. The habitat preference model was then validated using tracks of acoustically tagged free-ranging adults. The model predicted that ~9% of historically available habitat is now blocked by dams and that much of this habitat would have been suitable for spawning. Similarly, acoustic tracking of free-ranging green sturgeon adults has shown different migratory behaviour based on seasonal operation of Red Bluff Diversion Dam (RBDD) gates in the Sacramento River, suggesting upstream movement was hindered (Heublein *et al.*, 2009). Further, aggregations of green sturgeon near a water pumping facility (Glen Colusa Irrigation District pumping facility) suggest these facilities may influence spawning and post-spawn holding habitat and freshwater movements (Heublein *et al.*, 2009). Several other partial or complete barriers to migrating adult green sturgeon exist throughout the Californian Central Valley landscape. Under normal flows the Sunset Pumps Weir in the Feather River limits migration to upstream spawning habitat. Multiple reports of adult green sturgeon at the base of Daguerre Point Dam in the Yuba River infers this structure also blocks sturgeon from upstream spawning habitat (Heublein *et al.*, 2017). Upstream migrating sturgeon are also impeded by flood control structures in the Sacramento and Feather rivers. Adult green sturgeon are stranded near Fremont and Tisdale weirs in ponded water after flood flows recede on the Yolo and Sutter bypasses (Thomas *et al.*, 2013; Heublein *et al.*, 2017).

Decommissioning non-essential water reservoirs may provide improved habitat connectivity. Steel *et al.* (2018) compared migratory movements of acoustically tagged adult green sturgeon before and after the decommissioning of RBDD in the Sacramento River. Prior to decommissioning the RBDD the proportion of tagged fish migrating upstream past the dam was linked to gate closures. When gate closures were delayed by a month, significantly more green sturgeon migrated above the dam. Following dam decommissioning, the proportion of fish successfully reaching spawning habitat above the dam increased. These findings suggest the RBDD was blocking movement to spawning grounds and highlight that decommissioning of dams can be an effective management action to reconnect critical habitat.

#### Management actions and data gaps

The studies reviewed here present strong evidence to show that waterway infrastructure can obstruct green sturgeon movement. Water diversion entrainment likely represents a significant source of early-life mortality, particularly in outmigrating juveniles (Mussen *et al.*, 2014). Placing limits on water diversion inflow velocities according to time of day, season and location, may be effective in lowering entrainment rates (Verhille *et al.*, 2014), but validating these estimates under field conditions is required. Swimming performance estimates derived from non-volitional laboratory trials can sometimes underestimate true capacity, because fish can attain greater swimming speeds under open-channel, volitional conditions (Hinch and Bratty, 2000; Peake, 2004). Moreover, the swimming performance data were collected at a single test temperature (18.5°C), but the thermal sensitivity of swimming performance needs to be assessed to appropriately inform season-specific water operations. Assessing burst swimming performance, across a range of temperatures, may also provide useful information because maximal, anaerobic swimming efforts are likely employed by fish avoiding entrainment.

A suggested additional management action is to install fish-protection devices at water diversion pipes. Poletto *et al.* (2014b, 2015) offer several fish-protection device designs (protective screen, louvre box, trash-rack box and perforated cylinder), which significantly lowered entrainment risk in laboratory trials. Field-testing these designs under a range of conditions (e.g. manipulating turbidity, light and temperature) will provide a more detailed assessment of their effectiveness. Furthering our understanding of how fish interact with these protection devices may aid in creating a design where impingements are minimized. Constructing and maintaining fish-protection devices can be expensive, and ~98% of diversions in the S-SJ watershed remain unscreened (Herren and Kawasaki, 2001). Experimental assessments of fish protection devices will allow for the identification of the most effective designs that minimize impingements that are also cost effective (Moyle and Israel, 2005).

Water reservoirs present large-scale barriers to green sturgeon and may block access to critical spawning grounds. Decommissioning non-essential dams will likely prove effective in reconnecting aquatic habitat. In instances where decommissioning is unfeasible, delaying gate closures during peak spawning migrations may allow adults to reach preferred spawning habitats (Steel *et al.*, 2018). Additionally, the installation of fishways can greatly enhance passage prospects but designs accommodating the swimming capacity and behaviour of green sturgeon are still needed. Fish ladders designed for salmonids have been shown to be inappropriate for sturgeons (Cheong *et al.*, 2006; Kynard *et al.*, 2011), and green sturgeon may require a specific design to optimize passage (e.g. fishway slope, length and height). Taken together, the studies reviewed here highlight the vulnerability of green sturgeon movement to barriers, yet preliminary assessments of management strategies offer promising results.

## Conclusion

The data synthesis provided here allowed the identification of key data gaps and priority research areas. All experimental research to date has been conducted on the less threatened northern DPS and these findings may have limited applicability to the southern DPS because stressor impacts can differ between populations in a range of fishes (Eliason *et al.*, 2011). Gaining research access to southern DPS fish could prove invaluable to the protection and long-term persistence of green sturgeon. Studying the effects of stressors on early life-history stages must also be prioritized, because research has been biased towards juveniles and adults. Less than 10% of experimental studies examined the combined effects of stressors on green sturgeon physiology, despite the growing recognition that organisms are rarely exposed to stressors in isolation in natural settings. Future research efforts should aim to characterize interactions among both biotic (e.g. competition and predation) and abiotic stressors (e.g. elevated temperatures and hypoxia) to inform holistic management strategies which target co-occurring threats. Green sturgeon research has also centred on understanding drivers of population declines during the freshwater life history phase, and far less work has been directed towards assessing the impacts of stressors experienced during the marine life history phase, such as ocean acidification and fisheries bycatch. Lastly, several potential threats remain uninvestigated in green sturgeon, including exposure to a wide range of contaminants in their habitat (e.g. heavy metals and pesticides), predation pressure from invasive fishes and the impact of low dissolved oxygen levels (i.e. hypoxia) on their performance and survival.

Despite these data gaps, this meta-analytic review synthesized the existing research on green sturgeon and provided a quantitative assessment of the impacts of prominent stressors to identify potential management actions. Meta-analyses facilitate evidence-based conservation practices by providing a summary of relevant, empirical evidence required for management decisions (Cook *et al.*, 2014). By combining data from numerous sources, meta-analyses also generate greater explanatory power and may reveal effects not detected by individual studies (Mulrow, 1994; Gurevitch *et al.*, 2018). Here, we show that the use of meta-analyses can inform management decisions by synthesizing existing evidence and highlighting knowledge gaps. A wealth of literature on the ecophysiological constraints dictated by environmental stressors exists for many species of concern which can be summarized for species management guidelines. For example, several science-based management actions have been suggested here, such as, ensuring water temperatures are maintained within optimal ranges during peak spawning periods, mitigating salt intrusion in nursery habitats and placing limits on water diversion inflow velocities according to time of day, season and location.

Importantly, the meta-analytic approach implemented here can be used to synthesize ecophysiological data on a wide range of imperiled, keystone and ‘umbrella’ species. This approach can be extended to groups of focal species/species assemblages to reveal if stressors have generalizable effects within an ecosystem. Meta-analytical data can also determine the life-stage-specific thermal tolerance window of various species inhabiting a single waterway and management actions can target temperatures where the greatest thermal overlap occurs among species. Similarly, species-specific tolerance limits to contaminants can be determined using meta-analytics to establish lowest admissible contaminant concentrations based on the tolerance limits of the most sensitive species (O’Brien and Keough, 2014). The meta-analytic approach provided here offers an additional tool to inform conservation decisions, where a multitude of factors (e.g. economic, cultural or social value of a species, stakeholder opinion, urgency and logistics) are considered when deciding which species or habitat region to prioritize for conservation. In conclusion, quantitative syntheses can facilitate the integration of ecophysiological data into species management plans, identify knowledge gaps and direct future research actions, with the ultimate goal of maximizing conservation gains from research efforts.

## Funding

This work was supported by Delta Stewardship Council (grant #1470 awarded to N.A.F., A.E.T. and R.E.C.), the University of California, Davis Agricultural Experiment Station (grant #2098-H awarded to N.A.F and grant #2252-H awarded to A.E.T.) and the Department of Water Resources (grant #4600010855 awarded to N.A.F.).

## Supplementary Material

Supplementary_data_coz035

Supplementary_table_I_coz035
