# Supplementary material for: Integrating physiological data with the conservation and management of fishes: a meta-analytical review using the threatened green sturgeon (Acipenser medirostris)
Source: Conserv Physiol. 2019 Jun 28;7(1):coz035. doi: 10.1093/conphys/coz035 (PMC6601218; doi:10.1093/conphys/coz035)
Supplement: Supplementary_table_I_coz035 [file supplementary_table_i_coz035.docx]

**Supplementary material**

|  |  | **Regression test** | | | **Rosenburg's fail** | ***Trimfill* data** |
| --- | --- | --- | --- | --- | --- | --- |
| **Stressor** | **Trait** | **t** | **df** | **P** | **safe number** | **augmentation** |
| Elevated Temperatures | Growth | 0.05 | 2 | 0.96 | 2513 | 3 |
|  | Hsp | 0.35 | 9 | 0.74 | 489 | 4 |
|  | Deformities | 0.13 | 7 | 0.90 | 4439 | 2 |
|  | Hatching Success | -2.78 | 7 | 0.02 | 562 | 2 |
| Salinity | Growth | -1.29 | 4 | 0.27 | 12 | 0 |
|  | Plasma Osmolality | 1.82 | 16 | 0.09 | 1335 | 2 |
|  | Muscle Moisture | 0.88 | 9 | 0.40 | 377 | 1 |
| Food Restriction | Growth | -1.31 | 14 | 0.21 | 498 | 6 |
|  | Hsp | 0.44 | 14 | 0.67 | 72 | 0 |
|  | CTmax | -0.77 | 4 | 0.48 | 3 | 0 |
| Contaminants | Growth | -0.52 | 3 | 0.64 | 5 | 3 |
|  | Mortality | 0.06 | 5 | 0.96 | 167 | 0 |
|  | Whole-body Burden | 1.76 | 4 | 0.15 | 244 | 0 |

**Table I.:** Summary of statistical outputs from random effects models for measures of publication bias including regression test, Rosenberg’s fail safe number, and *trimfill* data augmentation.
